# Enteritis necroticans and *Clostridium perfringens* type C; Epidemiological and pathological findings over the past 20 years

**DOI:** 10.1371/journal.pntd.0012836

**Published:** 2025-02-05

**Authors:** Stuart Johnson, Andrew M. Skinner, Calob Lostutter, Trevor Duke, Horst Posthaus

**Affiliations:** 1 Edward Hines, Jr. VA Hospital, Hines, Illinois, United States of America; 2 Loyola University Medical School, Maywood, Illinois, United States of America; 3 University of Utah, School of Medicine, Salt Lake City, Utah, United States of America; 4 George E Wahlen VA Hospital, Research Service, Salt Lake City, Utah, United States of America; 5 Georgia-Pacific LLC, Atlanta, Georgia, United States of America; 6 Department of Paediatrics, University of Melbourne, Melboourne, Australia; 7 School of Medicine and Health Sciences, University of Papua New Guinea, Port Moresby, Papua New Guinea; 8 Institute of Animal Pathology, Vetsuisse Faculty, University of Bern, Bern, Switzerland; National Centre for Infectious Diseases, SINGAPORE

## Abstract

Enteritis necroticans (EN) in humans caused by infection with *Clostridium perfringens* type C, once thought limited to the highlands of Papua New Guinea has been identified sporadically worldwide. Outbreaks still occur among children in low-income countries and isolated cases occur among children and adults in other countries. Here the disease seems to be associated with diabetes mellitus and other risk factors. *C. perfringens* type C is also an important cause of necrotizing enteritis among animals, particularly pigs. Research into the pathogenesis of this disease has confirmed the central role of beta toxin and its target, the endothelial cell. Unlike most bacterial enteric infections, the primary anatomic location of EN is the proximal small intestine, reasons for which are not completely understood. Ongoing surveillance for *C. perfringens* type C infection is warranted as well as public health measures of prevention in locations where environmental and food hygiene is poor.

## Introduction

Enteritis necroticans (EN) was once endemic in the Highlands of Papua New Guinea (PNG), where it was called pigbel because of the typical abdominal presentation of the disease and its association with ritual pig feasts [1]. This disease caused by infection of a specific toxin-producing strain of *Clostridium perfringens* is unique in the anatomic location of the pathology, age distribution of those affected, and geographic distribution of cases [2]. While the disease may no longer be highly prevalent in PNG, cases have been reported worldwide. The etiologic agent, *C. perfringens* type C, is widely distributed and responsible for very similar diseases in animals and humans [3]. EN probably dates back to antiquity and there is even speculation that Siddhartha Guatama (the Buddha) died from pigbel after eating a meal of tainted pork [4]. We summarize data published over the last 20 years in humans, epidemiology of EN in PNG and outside of PNG, other related clostridial diseases, and discuss the current knowledge about the pathogenesis of *C. perfringens* type C enteritis in humans and pigs.

## Methods

A literature search from January 1, 2000 to October 1, 2023 was conducted using electronic databases (PubMed, Scopus, and Global Health). Reference lists from extracted articles or reviews were also searched. The following search terms were included: pigbel, pig-bel, “pig bel”, enteritis necroticans, *Clostridium perfringens* type C, and beta toxin. Non-English reports were included if an English version of the abstract or summary was available. We searched the literature for studies describing reports of EN in humans, studies on the etiologic agent, beta toxin-producing *C. perfringens*, type C, and animal diseases linked to *C. perfringens* type C. Studies that reported diseases not associated with *C. perfringens* type C, such as necrotizing enterocolitis of newborns were excluded.

### History of enteritis necroticans

Enteritis necroticans (EN) first received attention after World War II when it emerged in Germany and other European countries and was called *Darmbrand* or *‘fire bowels’.* This disease was notable for severe abdominal pain, nausea and vomiting and was often fatal. *Clostridium perfringens* (previously, *C. welchii*) isolates were recovered from necrotic intestinal tissue and the disease was thought to be related to toxins produced by these clostridia [5]. Seven of these Darmbrand-associated strains were recovered from storage cultures and characterized by modern genotypic and phenotypic analyses [6]. Five of the strains were confirmed as type C and carried genes for beta toxin (*cpb*) and enterotoxin (*cpe*) on large plasmids. All seven produced highly heat-resistant spores and shared spore heat-resistant mechanisms with *C. perfringens* strains that cause human food poisoning. There was evidence supporting food-borne transmission, but malnutrition and other factors may have also been involved in the pathogenesis of *Darmbrand* [7]. The disease disappeared in the 1950s possibly because of the overall improvement in sanitation and general nutrition of the population. Unlike cases in PNG; however, the cases in Germany affected primarily people 30–50 years of age [7].

The first case of pigbel in PNG was reported in 1961 and was thought to be caused by the consumption of contaminated pork. The pig in Papua New Guinean culture is of social and dietary importance. Pigs were kept in close contact to humans and were used for ceremonial practices where they were eaten to celebrate rite of passages—the most notable being called “Pig Feasts”. The exact feast preparation varies by PNG region [7], but standard preparation includes butchering it publicly (oftentimes large numbers of pigs), shortly roasting the pig over an open fire to singe off the hair, and then cooking it over several hours in ground pits heated with hot stones. Samples of soil from the highland villages in which these rituals take place have identified *C. perfringens* type C by fluorescent antibody staining [8]. *C. perfringens* type C isolates were recovered in the intestines of patients in PNG exhibiting pigbel [9] suggesting these cultural practices compounded with an otherwise low-protein diet apart from pork at ritual feasts provide a route of infection and predisposition for pigbel [10]. It was also postulated that consuming sweet potato, a major staple of the traditional diet among highlanders, played a role in the disease. Sweet potatoes contain trypsin inhibitors that could potentially suppress pancreatic enzyme activity that aids in breakdown of the beta toxin leading to the proliferation of the toxin and subsequent necrosis of the jejunal and ileal mucosa [2]. Evidence from multiple sources supports the role of trypsin inhibitors in the pathogenesis of this disease including experiments where guinea pigs were challenged intragastrically with *C. perfringens* type C and raw soybean flour developed pigbel-like lesions but not when challenged with the pathogen alone or when the soybean flour was autoclaved destroying the trypsin inhibitors [11]. Trypsin inhibition is a particularly important risk factor given the central role of the trypsin-sensitive beta toxin in the pathogenesis of EN. However, host factors other than a diet replete with trypsin inhibitors may also contribute to susceptibility including malnutrition and chronic diseases such as diabetes, particularly outside of PNG.

From time of the first reported cases in early 1960s to the 1980s, pigbel reached epidemic significance in the highlands of PNG and was cited as the second leading cause of death in children over the age of 1 [11]. Children aged 2–10 years old were more at risk of infection, while males were affected more than females. Among those, 16–20 aged females were more likely to contract the disease compared to men, possibly explained by marriage ceremonies involving pig feasting where the bride consumes large amounts of pork to encourage fertility [7].

Pigbel is clinically characterized by acute abdominal pain and is often accompanied with bloody diarrhea, vomiting blood [12], and bloating of the stomach due to upper abdominal distension—where the name Pigbel (pain in belly associated with ritualistic pig-feasting) was coined [9]. Death of affected patients can occur within 24 h after onset of clinical signs due to enteric necrosis progressing to peritonitis and/or septic shock [9,11]. Macroscopic intestinal lesions are characterized by segmental hemorrhage and necrosis of mainly the jejunum. Lesions can progress to a full thickness necrosis in small intestinal segments, and also extend to the large intestine. Histopathological hallmarks in acute cases are deep mucosal necrosis with vascular thrombosis and necrosis accompanied by extensive hemorrhage. Subacute lesions are more often described as multifocal patchy necrosis of the small intestine.

A toxoid vaccine targeting *C. perfringens* type C was introduced in 1979. A widespread vaccination program for children in the highlands led to a dramatic decrease in pigbel cases [13]. Pigbel was then thought to be no longer endemic to PNG and disappeared as it did in Europe in the 1950s. With production of the vaccine ceasing in 1992 and the remaining stock used or discarded the vaccination program also ceased. However, recent studies from 2006 to 2015 have documented confirmed pigbel cases in Highland hospitals and there are potentially more cases in areas where surveillance is more difficult [1,14].

### Epidemiology of enteritis necroticans in Papua New Guinea post-vaccination program

Prior to vaccination in areas where pigbel prevalence was high in 1979, Goroka Hospital in the Eastern Highlands Province reported more than 100 cases annually of children admitted with pigbel while Mount Hagen Hospital in the Western Highlands Province reported 60–100 cases annually. Post-vaccine era numbers from 1993 to 2001 demonstrate a marked decrease of reported cases of pigbel with cases reported less than 5% of pre-vaccination numbers in Goroka and less than 10–20% of pre-vaccination numbers in Mount Hagen [15]. At the start of the immunization program in 1981, Tari Hospital (Enga Province) reported that the number of deaths related to pigbel among children under 5 years of age had dropped from 36 to 5 within 2 years of administration.

Cases of pigbel were still being diagnosed in PNG after the vaccination had ceased to be administered. From 1999 until the end in 2001, the PNG National Health Information System (NHIS) reported 105 pigbel cases from hospitals in the Southern Highlands, Simbu, Eastern Highlands, Western Highlands, and Enga Provinces [15].

In a study conducted in 2002, 119 children with acute abdominal pain were identified in 38 health centers and hospitals in the highlands over the 12-month period. Nine percent were confirmed cases of pigbel and 16% were identified as probable EN. Of the confirmed and possible cases, 50% were found to cluster along the border of the Western Highlands and Enga Provinces [15].

A surveillance study was piloted for pigbel based on a standardized clinical case definition and a beta-toxin immunoassay (EIA) for testing of intestinal fluid in suspected cases from 2012 to 2015 [1]. This proof of concept trial was conducted at one hospital in the Jiwaka Province [14]. Inclusion criteria for the study included patients admitted to the hospital with severe abdominal pain of less than 2 weeks duration. A standardized case definition was used to identify likely cases of pigbel and patient specimens including gastric aspirates, stool, and intestinal fluid were tested for the presence of *C. perfringens* beta toxin by immunoassay (EIA). Among 105 patients with acute abdominal pain, 48 met the case definition. The mean age was 5 years (IQR: 2–6) and 35% (12/34) tested positive for beta toxin. Four patients who did not meet the case definition were also positive by the beta-toxin EIA and four with classical pathologic lesions tested negative by the EIA. Five children died, four of whom met the case definition. The EIA has not been subsequently used for resource reasons.

Between 2013 and 2022, the Paediatric Hospital Reporting program, a nation-wide surveillance program for all childhood hospital admissions, recorded 113 cases of pigbel and 14 deaths, diagnosed based on the standardized and/or surgical clinical criteria. Annual cases are listed in Table 1. This reporting system involves 16–24 hospitals, but not all are able to report a complete set of pediatric data each year. Additional epidemiologic data such as association with pig feasts or specific food consumption are not available from this program, but there is speculation that many of these cases are sporadic and not associated with feasts or large gatherings.

**Table 1:** Annual Pigbel cases and deaths reported to the Papua New Guinea Paediatric Hospital Reporting program: https://pngpaediatricsociety.org/reports/annual-child-morbidity-and-mortality-reports/.

Year	Pigbel cases	Deaths
2013	18	2
2014	20	3
2015	3	1
2016	33	1
2017	11	4
2018	11	1
2019	3	0
2020	7	1
2021	2	0
2022	5	1
Total	113	14

In conclusion, there is an ongoing burden of pigbel in PNG. Beta-toxin EIA testing is helpful for disease confirmation; however, the test is insensitive and a negative test does not rule out pigbel.

### Epidemiology of enteritis necroticans outside of Papua New Guinea

Five reports of EN were identified in the literature since 2000 where *C. perfringens* type C was recovered from intestinal specimens or was confirmed present based on amplification of the *cpb* gene from involved tissue (Table 2). Four of these reports represented individual cases and the other report was a summary of an outbreak in eastern Sri Lanka where 42 patients were seen in a 6-month period in 2002 [16]. Two-thirds of the cases in this outbreak were younger than 10 years of age, laparotomy identified small intestinal lesions consistent with EN in 13 patients, and *C. perfringens* was recovered in 7, with *C. perfringens* type C confirmed by PCR typing of the isolates in 4. The four individual case reports gave more detailed epidemiologic and clinical findings and represented patients from the United States of America (USA) (two cases), Japan, and the United Kingdom (UK) [17–20]. These cases ranged in age from 12 to 66 years, three were male, and diabetes mellitus (DM) was a co-morbidity in three of the four. All four patients had consumed a food source prior to symptom onset that has been historically associated with other forms of food poisoning; Chitterlings, locally processed turkey sausage, raw seafood, and chicken. Three of the four patients recovered after surgical resection of the involved necrotic small intestinal segments.

**Table 2:** Enteritis necroticans (confirmed or presumed *Clostridium perfringens* type C associated) reported outside of Papua New Guinea since 2000.

Location, yr	Age/Sex	Underlying illness	Confirmatory evidence	Additional comments	Outcome	Reference
*C. perfringens* type C or beta toxin confirmed						
USA (Georgia), 1998	12/Male	Diabetes mellitus	Extensive jejunal necrosis; PCR amplification of *cpa* and *cpb* genes from resected tissue.	Chitterlings (pig intestines) ingested 48–72 hr prior to onset	Recovered after jejunal resection	[17]
USA (Mississippi), 2002	66/Female	Diabetes mellitus	Segmental necrosis of the ileum and jejunum; *C. perfringens* type C identified by PCR amplification of cpb gene from jejunum	Locally processed turkey sausage was presumed source of infection	Died	[18]
Japan (Sapporo), 2005	50/Male	Diabetes mellitus	Segmental annular mucosal lesions; immunohistology staining of mucosa positive for *C. perfringens* beta-toxin	Meal of raw scallops, salmon, crab prior to symptoms	Recovered after 70 cm of jejunum resected	[19]
Sri Lanka (Batticaloa), 2002	42 cases (66% <10 years age)		Lapatoromy in 13 patients showed segmental and circumferential inflammation of the small intestine; histology showed patchy hemorrhagic infiltration, necrotic zones with intervening segments of normal intestine; *C. perfringens* recovered in 7 cases (4 confirmed a type C by typing)	Classified cases as Highly acute (n, 7) Acute surgical (n, 10) Subacute surgical (n, 2) Mild or moderate (n, 24)	Five of seven cases with highly acute form died; All 10 cases with acute surgical form had laparotomy and survived	[16]
UK (London), 2016	54/Male	Previously healthy	Necrosis of ileum and proximal colon*; C. perfringens* type C recovered from resected bowel	Possible chicken curry source; A mechanically obstructing diverticular stricture may have contributed to syndrome by delayed intestinal transit	Recovered after subtotal colectomy and long-segment ileal resection	[20]
*C. perfringens* type C suspected						
India (New Delhi), 1992–1998	18 cases of acute jejunoileitis; mean age 6.5 years (range 6 mo–12 yr); Male:Female ratio 1.2:1	No evidence for chronic malnutrition	Patchy transmural mucosal necrosis extending centrifugally with submucosal edema, interstitial hemorrhage	Authors suspected process was “immune mediated”	All patients underwent laparotomy and the’10 patients with complete resection of the involved bowel had dramatic improvement after surgery	[21]
USA (Louisiana), 2002	53/Female	Diabetes mellitus	Transmural necrosis of duodenum on autopsy; *C. perfringens* recovered from peritoneal fluid	Findings also suggestive of ischemic enterocolitis	Died	[23]
USA (Missouri), 2004	3/Male	Cyclic neutropenia	Necrotic proximal jejunum and distal ileum; PCR amplification of *cpa* gene from tissue	Evidence for *C. perfringens* infection, but type C not confirmed	Recovered after resection of jejunal and ileal segments	[64]
Bangladesh (Chittagong), 2004–2009	24 cases of acute abdomen; mean age 5.5 years (range 6 mo–12 yr); 58% males		Segmental enteritis involving the jejunum (17), jejunum and ileum (5) or ileum alone (2); scattered dark patches on intestinal wall (16), circumferential dusky lesions (4), perforation (1) or frank gangrene (3)	“*C. welchii*” not detected in peritoneal fluid, but tissue cultures not performed and microbiologic methods not elaborated	All patients underwent laparotomy, 4 underwent bowel resection, 2 died	[22]
Belgium, 2018	42/Male	Previous partial colectomy for volvulus	Necrotic jejunum; No microbiologic studies performed		Recovered after resection of jejunal segment	[24]
Switzerland, 2019	70/Male	Bariatric surgery 30 years previously	Megacolon with portal venous gas; necrotic small intestine; *C. perfringens* recovered from intraoperative specimen		Outcome not reported after resection of necrotic small intestine and colon	[25]
Australia, 2022	15/Female	Recent bone marrow transplant	Extensive hemorrhagic necrosis of small bowel with pneumatosis intestinalis and periportal pneumatosis in the liver	*C. perfringens* recovered from blood and peritoneal fluid	Died	(This review)

**Fig 1:**
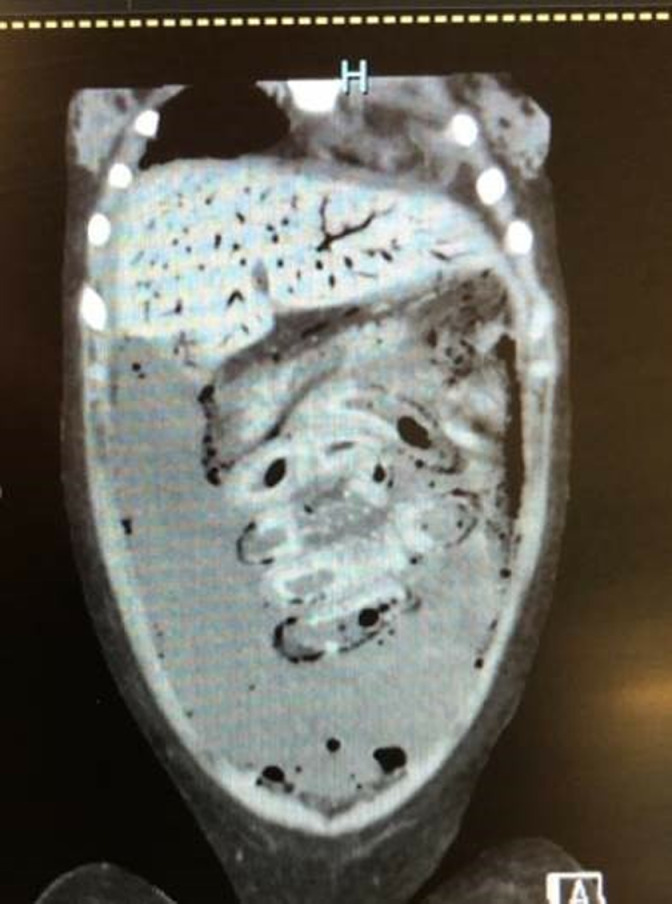
Abdominal CT scan of a 15-year-old girl with abdominal sepsis 1 week after a bone marrow transplant. Hemorrhagic necrosis of the small bowel is seen in addition to extensive pneumatosis intestinalis, ascites and the periportal pneumatosis in the liver. *Clostridium perfringens* was recovered from the blood and ascitic fluid.

In addition to the above-confirmed reports, seven additional reports of EN were identified where *C. perfringens* type C was suspected, but not confirmed by microbiological or molecular testing (Table 2). Five of these reports represented individual cases and two reports were case series from the Indian subcontinent. One of these case series described 18 laparotomy-confirmed cases of EN in children aged 6 months to 12 years over a 6-year period from New Delhi, India [21]. They documented patchy transmural mucosal necrosis of the jejunum (primarily) and ileum and the 10 patients that had complete resection of the involved intestine showed dramatic improvement following surgery. The authors also noted that many more cases were suspected clinically, but responded to conservative management. The second case series described segmental enteritis in 24 children over a 6-year period from Chittagong, Bangladesh [22]. All cases underwent laparotomy and showed predominantly jejunal necrotic lesions. The five individual case reports involved three males and two females, ranging in age 3–70 years, from the USA (two cases), Belgium, Switzerland, and Australia [23–25]. Potential predisposing factors included DM, cyclic neutropenia, and previous bowel surgery (two cases). One case, in a child with immunodeficiency, developed extensive hemorrhagic necrosis and pneumatosis of the entire small bowel one week after a bone marrow transplant (Fig 1). All cases involved necrotic sections of small intestine, and *C. perfringens* was identified in four of the five cases, but *C. perfringens* type C was not confirmed.

In addition to clinical cases of EN, *C. perfringens* type C has been isolated from numerous clinical and environmental samples worldwide including stools of infants in Jordan [26], fresh water fish in China [27], farm animals in Saudi Arabia [28], horse fecal and soil samples from South Korea [29], and soil samples from the USA [30,31].

In summary, reports of EN from South Asia since 2000 describe cases similar to those reported in PNG and include primarily young children with similar frequency among males and females and with clinical presentations that varied in severity. In contrast, case reports of EN from the USA, Europe, and Japan describe a wide age range of affected patients who were predominately male (6 of 9 case reports). DM is a common predisposing risk factor (4 of 9 cases) and all of the confirmed cases outside of the Indian subcontinent describe a potentially contaminated meal eaten prior to symptom onset. The onset of symptoms after the meal in most cases (1–2 days) suggest infectious enteritis rather than ingestion of preformed toxin.

### Pathogenesis of enteritis necroticans

*C. pefringens* type C has early on been identified as the cause of EN due to the epidemiological evidence of its association with the disease in humans. The pathogen also causes similar enteritic disease in horses, sheep, goats and cattle, but the pig is the by far most frequently affected host species (Table 3). Much of our knowledge about the pathogenesis of human EN is extrapolated from studies on naturally occuring *C. perfringens* type C enteritis in animals, ex vivo laboratory work and research using experimental animal models. The pathogenesis of *C. pefringens* type C in pigs is reviewed elsewhere [3]. Here, we relate the most noteworthy parallels to the human disease.

**Table 3:** Enteric disease in animals due to *Clostridium perfringens* type C.

Species	Clinical Manifestations	Predisposing factors	Pathology	Outcome	Prevention	Reference
Piglets	Peracute: sudden death within 3 days after birth; Acute: Hemorrhagic diarrhea and death, usually during first week of life; Subacute/chronic: Non-hemorrhagic diarrhea, depression, emaciation, death 2–3 weeks post-partum	Environmental contamination Asymptomatic carriers	Peracute to acute form: segmental transmural hemorrhagic necrosis in jejunum, occasionally extending to ileum and colon) with mucosal and submucosal thrombosis Subacute form: segmental jejunal mucosal necrosis demarcated by degenerated neutrophilic granulocytes	Fatal	Vaccines Sanitation	[3,65,66]
Cattle	Onset day 1 to 1 month. Symptoms include emaciation, anorexia, dehydration and hemorrhagic diarrhea	No data	Segmental —transmural hemorrhagic necrosis of the jejunum and ileum, occasionally involving the reticulum, rumen, spiral colon and cecum	Fatal	Vaccines Sanitation	[66,67]
Horses	Onset day 1–5 months Acute onset of diarrhea, colic or sudden death. Primarily impacts foals, but not limited	No data	Segmental or diffuse, transmural hemorrhagic necrosis of the jejunum and ileum, but also involving the colon and cecum with mucosal and submucosal thrombosis	Fatal	Vaccines Sanitation	[29,68]
Sheep/Goats	Hemorrhagic enteritis in neonates and occasionally adult sheep	No data	Segmental or diffuse, transmural hemorrhagic necrosis of the ileum;	Fatal	Vaccines Sanitation	[66,69]

Type C strains are defined by secretion of alpha- and beta-toxin [32], but they secrete a plethora of additional toxins and enzymes. Whereas alpha-toxin (a phospholipase) is produced by all *C. perfringens* strains, including apathogenic strains, beta-toxin is unique to type C and B strains. Using animal models and genetic modification of *C. perfringens* type C strains it was shown that beta-toxin was the essential virulence factor for induction of enteric lesions [33].

**Fig 2:**
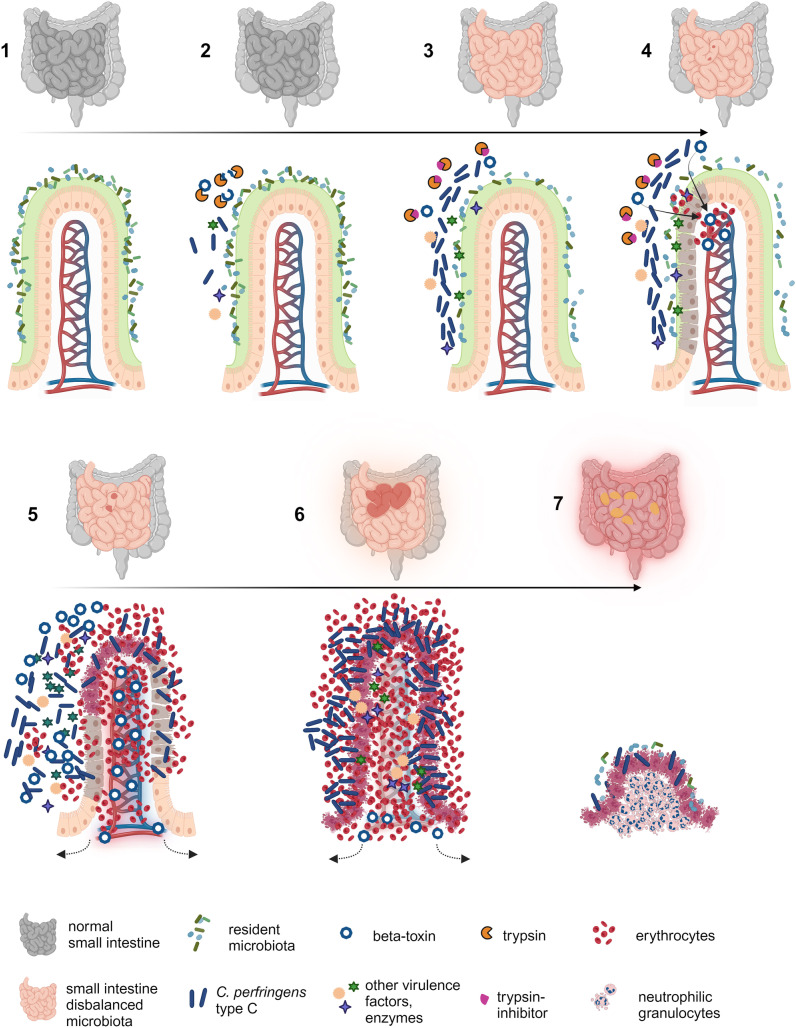
Proposed pathogenesis of enteritis necroticans. (1) Healthy intestine with microbiota; (2) colonization with *Clostridium perfringens* type C, beta-toxin degradation through trypsin; (3) proliferation and toxin secretion of *C. perfringens* type C, potentially due to disbalanced microbiota, uptake of large numbers of pathogen, trypsin inhibitors preventing beta-toxin degradation, or other factors that may facilitate proliferation and toxin elaboration; (4) initial epithelial barrier alteration (through additional clostridial virulence factors or other co-pathogens), diffusion of beta-toxin into lamina propria, local endothelial damage with plasma extravasation and small hemorrhages, lesions macroscopically most likely not yet visible; (5) increased nutrient supply for *C. perfringens* through vascular leakage, increased proliferation and upregulation of toxin secretion leads to acceleration of damage, lesions become macroscopically visible and extend rapidly; (6) vicious cycle of clostridial proliferation, toxin secretion, vascular damage and nutrient supply leads to rapidly progressing hemorrhagic transmural jejunitis with peritonitis, lesions potentially extend into distal small and also large intestine, death can occur at this stage; (7) in more protracted cases patchy lesions develop and there is marked inflammatory response in affected intestinal segment, lesions become macroscopically less hemorrhagic and more fibrinous, severe peritonitis can develop. *Figure created with BioRender.com.*

**Fig 3:**
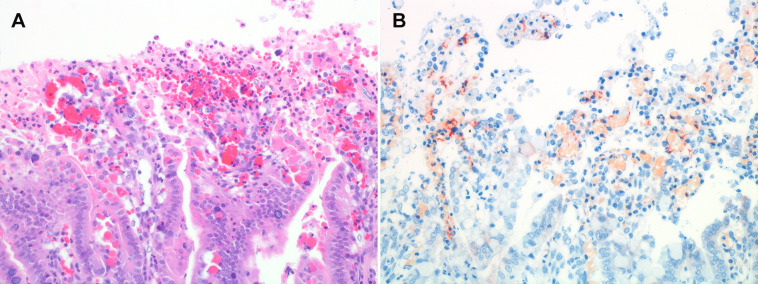
*Clostridium perfringens* beta-toxin targets endothelial cells of mucosal vasculature during early stages of lesion development in an experimental porcine infection model. **(A)** Histopathology of an early jejunal lesions of necrotic enteritis induced by *C. perfringens* type C in a porcine ileal loop model showing hemorrhage in the tips of villi and epithelial cell sloughing. Magnification 400× **(B)** Immunohistochemistry for beta-toxin of a serial section from A. Note that beta-toxin signal (red) stains the vascular outline (endothelial cells) in the lamina propria. (Samples derived from study by Schumacher and colleagues [70]).

Generally, *C. perfringens* spores or vegetative bacteria have to be orally ingested and germinate to colonize the intestine (Fig 2). This is followed by a phase of rapid proliferation, either immediately after colonization or in the case where favorable nutritional conditions occur. During their exponential growth phase, pathogenic type C strains secrete large amounts of toxins as well as enzymes and produce metabolites which as a whole can have deleterious effects on the host intestinal barrier [34]. Currently, the initial steps leading to an alteration of the small intestinal epithelial barrier are incompletely understood. Epithelial cells are resistant to beta-toxin [35–37], thus other virulence factors and/or the presence of resident co-pathogens may be important for this. For example enterotoxin, another virulence factor of *C. perfringens* capable of causing damage to epithelial tight junctions, is encoded by several *C. perfringens* type C strains including the Darmbrand-associated strains mentioned previously [5,6]. Epithelial barrier damage most likely allows beta-toxin to diffuse into the intestinal mucosa and reach the vascular endothelium (Fig 3). Endothelial cells and potentially platelets and immune cells are targeted via the specific interaction of the toxin with its membrane receptor Platelet Endothelial Cell Adhesion Molecule 1 [38–40]. Beta-toxin forms oligomeric transmembrane pores in the plasma membrane of target cells leading to endothelial cell necrosis and acute vascular damage. The result is acute local hemorrhage and further hypoxic damage in the affected part of the small intestinal mucosa [35,41–43]. Upon exposure to such nutrients, *C. perfringens* type C proliferates even further and upregulates toxin secretion [44]. This leads into a vicious cycle of bacterial proliferation, toxin secretion, beta-toxin-induced vascular damage, hemorrhage and clostridial toxin and enzyme-induced tissue damage, ending up in rapidly progressive intestinal necrosis. Toxemia, resulting from resorption of beta and other toxins through the damaged intestinal barrier most likely contributes to the disease in later stages; however, systemic effects of specific toxins have so far not been conclusively demonstrated in naturally occurring disease.

In case of human EN, it is not clear how and when during the disease process intestinal colonization occurs. *C. perfringens* spores can persist in the environment for years and the bacteria are believed to persist in the intestines of suitable hosts, such as pigs [45]. This can lead to long-lasting contamination of the environment and recurrent exposure of humans in endemic areas. This was most likely the case in the Highlands of PNG, where in the past, pigs were kept in very close contact to their human owners. The most prominent risk factor for humans in PNG in the past was the sudden consumption of pork meat following a traditional pig feast. Contamination of pork meat during the traditional slaughtering and cooking process, followed by distribution of the meat over a period of more than 1 day could have led to ingestion of large amounts of vegetative clostridia and/or spores. In addition, traditional pig feasting represented a sudden dietary change from a mainly vegetable to a meat-dominated diet, which could have affected the intestinal microbiota and favored clostridial overgrowth. Such sudden food changes are well-known risk factors for different clostridial enteric diseases in animals [46]. During pig feasting in PNG, newborn children still breast feeding were unlikely to be exposed to pork meat, but children 2–10 years of age were at highest risk to develop EN [7].

Another unproven factor might be the presence of resident co-pathogens that breach the upper intestinal epithelium, such as parasitic infection by *Strongyloides* or *Ascaris*. The adult forms of these parasites reside in the small intestine and were common among Khmer children with EN [47]. Compared to the disease in pigs, parasitic co-infections are not required for the development NE. However, *Isospora suis* invading and damaging small intestinal epithelial cells in newborn piglets, can initiate lesions that allow clostridia access to damaged tissue. Epithelial damage by coccidia (*Eimeria* sp.), are indeed an important predisposing factor in a very similar disease in chickens, caused by *C. perfringens* type G strains that produce NetB, a hemolysin beta-pore forming toxin related to beta-toxin [48]. In addition to providing a potential breach in the small intestinal epithelial barrier, adult parasites, such as *Ascaris* sp. are known to produce typsin inhibitors which are thought to facilitate their survival in a protease rich environment, but also may inhibit beta-toxin degredation [49].

The circumstances under which the more recent sporadic cases of EN occurred in PNG and elsewhere; however, are unclear. Association of sporadic cases with traditional pig feasts or sudden dietary changes is not consistently reported. With the currently available data, it seems likely that a common feature of EN is ingestion of contaminated food potentially in combination with dietary changes or other contributing factors facilitating *C. perfringens* type C proliferation and beta-toxin elaboration. Many human cases show pathological lesions resembling a more protracted course of the disease that is also observed in piglets dying of NE in the second or third week of life [50]. This could be due to either lower infectious doses ingested by the individual patient/animal, partial protection by the resident microbiota, or, a combination of both.

One important contributing factor in NE is that the presence of trypsin inhibitors in the small intestine. Beta-toxin is highly sensitive to degradation and inactivation by the pancreatic enzyme trypsin [51]. Thus, trypsin inhibitors can enhance stability and toxicity of beta-toxin [52]. In the highlands of PNG, sweet potato is the dietary staple and this is rich in trypsin inhibitors. One study showed that after 1 year of age PNG highlands children from areas of high risk for EN had significantly lower trypsin stool levels than controls including coastal village children, European or PNG children consuming “western” diets, and children under age 1 from high-risk area who were breast fed [53]. The association of NE with decreased trypsin activity is also paralleled in *C. perfringens* type C-induced enteritis in suckling piglets where sow colostrum contains high amounts of trypsin inhibitors [54].

DM has a striking association with EN cases outside of PNG and was documented in 4 of the 9 recent cases in our review. The reason for this association is not known, and while exocrine pancreatic dysfunction with trypsin/protease deficiency is a potential complication of type 1 DM, both types 1 and 2 DM appear to be associated [55]. DM is also associated with reduced gastric and small intestinal motility. A delay in small-intestinal transit time and overgrowth of bacteria in the proximal small bowel might also facilitate *C. perfringens* type C proliferation [56,57]. Further research is needed to elucidate the mechanism(s) whereby DM confers a risk for EN.

### Other human clostridial diseases related to enteritis necroticans

Over the past two decades, it has become apparent that other bacteria can cause necrotizing enteric diseases in humans, particularly *C. perfringens* strains that do not produce beta-toxin. They are referred to as *C. perfringens* type A in the literature, but some were shown to produce enterotoxin and they would now be referred to as type F strains [32]. These *C. perfringens* strains have been implicated in several necrotizing enteric disease syndromes sometimes closely mimicking *C. perfringens* type C-induced EN [58–60]. Sobel and colleagues described four adult patients in North America with necrotizing enterocolitis, three of them died, in whom *C. perfringens* was isolated or where clostridial antigens were detected by immunohistochemistry in affected intestinal specimens [60]. Cases were complicated by portal or mesenteric thrombosis in three patients and the same enterotoxin-producing *C. perfringens* strain was recovered from one patient, his wife, and food from a restaurant they ate at. The site of necrosis was the colon or distal ileum in three; the entire small bowel and colon in one, and the jejunum only in one case.

Another syndrome involving necrotizing colitis among institutionalized adults with chronic constipation has been linked to food borne enterotoxin-producing *C. perfringens* strains [61,62]. Bos and colleagues described a cluster of seven cases, two of them died, among residents at a residential care facility for mental illness in Oklahoma [61]. The necrotizing lesions involved the transverse, descending or sigmoid colon and *C. perfringens* isolates were recovered that contained a chromosomal cpe gene supporting food borne origin of the infection. A Thanksgiving meal containing turkey was the suspected vehicle. Constipation due to anticholinergic effects of psychiatric medications and fecal impaction were suggested as risk factors. Another report involved 42 residents and 12 staff at a Louisiana state psychiatric hospital who developed acute gastrointestinal symptoms [62]. Three of the residents, all who received medications with anti-intestinal motility side effects, died. Necrotizing colitis was documented post-mortem in two cases. *C. perfringens* enterotoxin (CPE) was detected in the stool of 20 of 23 ill residents and a chicken dinner consumed the previous day was associated with illness. CPE+ *C. perfringens* strain was also recovered from samples of chicken.

In summary, despite the lack of beta toxin, other *C. perfringens* strains have been shown to cause disease similar to *C. perfringens* type C-induced EN. Although not proven, predisposing factors may also dictate the location of the lesions such as constipation leading to colonic necrosis in some patients with food borne *C. perfringens* strain infection and co-infection with parasites leading to small bowel disease in patients with *C. perfringens* type C.

### Prevention of enteritis necroticans

To date, the most effective preventive method against EN caused by *C. perfringens* type C is vaccination. The human toxoid vaccine used from the late 1970s to 1990s in PNG was produced from formalin inactivated supernatants of *C. perfringens* type C strains and as such contained many secreted factors and exotoxins, including beta-toxin. As mentioned above, the vaccine campaign was remarkably successful, but discontinued after case numbers had dropped and remained low. The vaccine was administered mainly to the risk group of young children [13].

Similar vaccines are still widely and successfully used in veterinary medicine against different clostridial enteric diseases. (https://www.msdvetmanual.com/digestive-system/intestinal-diseases-in-pigs/clostridium-perfringens-type-c-enteritis-in-pigs#Treatment-and-Control_v3264050). Depending on the age of animals at risk, vaccines are used to induce protective immunity of the potentially affected animal itself (e.g., *C. perfringens* type D vaccines in sheep), or, in the case of pigs, to induce protective antibody levels in the colostrum of sows. These are then passively transferred to the piglets in the first days of life and provide sufficient maternal immunity to prevent disease outbreaks during the critical period of up to 3 weeks post-partum [63].

As *C. perfringens* vegetative forms or spores can be taken up by humans from the environment and contaminated food, standard hygiene measures and proper food processing are potentially the most important prophylactic measures against EN as well as the much more common enteric and other infections that lead to malnutrition, stunting, and chronic illnesses. Continued epidemiologic surveillance of EN and targeted investigations may clarify other risk factors such as sudden dietary changes, ingestion of contaminated foods, and the role of trypsin, and thus point to other preventive measures.

## Conclusions

EN continues to be reported sporadically in the highlands of PNG and worldwide. The etiology, *C. perfringens* type C is widely distributed, and also causes NE in livestock animals, mainly pigs. Over the past 20 years, significant new insights into the pathogenesis of this disease and the role of beta-toxin have been made. Future research needs to include comparative aspects between human and animal *C. perfringens* type C isolates, identifying risk factors for EN in humans, practical means of prevention and potential therapeutic interventions.

## Key learning points

- Enteritis necroticans in humans and a similar disease in animals, necrotizing enteritis, are caused by *Clostridium perfringens* type C which is worldwide in distribution.
- The disease typically presents as full thickness, segmental necrosis of the proximal small intestine and manifests as abdominal pain, bloody diarrhea and vomiting with high-associated mortality.
- Beta toxin, produced by *C. perfringens* type C is the essential virulence factor and the primary cellular target is the endothelial cell.
- The disease once endemic in Papua New Guinea has been reported elsewhere in South Asia as well as in North America, Europe, and Japan where there is a different age distribution and different associated risk factors.
- The disease is preventable by toxoid vaccination, but standard hygiene and proper food preparation are most important and more practical for prevention of human cases.

## Five key papers

1. Duke T, Myers S, Radcliffe J, Poka H, Deuel K, Miller CM, Pavlin BI. 2019. A surveillance system for pigbel in Papua New Guinea, based on a clinical case definition and laboratory confirmation. P N G Med J 62:114–121.
2. Lawrence GW, Lehmann D, Anian G, Coakley CA, Saleu G, Barker MJ, Davis MW. 1990. Impact of active immunisation against enteritis necroticans in Papua New Guinea. Lancet 336:1165–7.
3. Sayeed S, Uzal FA, Fisher DJ, Saputo J, Vidal JE, Chen Y, Gupta P, Rood JI, McClane BA. 2008. Beta toxin is essential for the intestinal virulence of *Clostridium perfringens* type C disease isolate CN3685 in a rabbit ileal loop model. Mol Microbiol 67:15–30.
4. Bruggisser J, Tarek B, Wyder M, Muller P, von Ballmoos C, Witz G, Enzmann G, Deutsch U, Engelhardt B, Posthaus H. 2020. CD31 (PECAM-1) Serves as the Endothelial Cell-Specific Receptor of *Clostridium perfringens* beta-Toxin. Cell Host Microbe 28:69–78 e6.
5. Wormald JC, Dindyal S, Mellor F, Behar N. 2016. Adult necrotising enterocolitis-pig-bel disease: a Pacific disease in London. BMJ Case Rep 2016.

## References

[1] Duke T, Poka H, Myers S, Radcliffe J, Pavlin BI. Pigbel in the 21st century: still here, and still in need of an effective surveillance system. P N G Med J. 2013;56(3–4):136–40. Epub 2013/09/01. 26288931

[2] Murrell TG, Walker PD. The pigbel story of Papua New Guinea. Trans R Soc Trop Med Hyg. 1991;85(1):119–22. Epub 1991/01/01. doi: 10.1016/0035-9203(91)90183-y 2068739

[3] Posthaus H, Kittl S, Tarek B, Bruggisser J. Clostridium perfringens type C necrotic enteritis in pigs: diagnosis, pathogenesis, and prevention. J Vet Diagn Invest. 2020;32(2):203–12. Epub 2020/01/21. doi: 10.1177/1040638719900180 ; PMCID: PMCPMC7081500 31955664 PMC7081500

[4] Chen TSN, Chen PSY. The death of Buddha: a medical enquiry. J Med Biogr. 2005;13(2):100–3. doi: 10.1177/096777200501300208 19813312

[5] Zeissler J, Rassfeld-Sternberg L. Enteritis necroticans due to Clostridium welchii type F. Br Med J. 1949;1(4597):267–9. Epub 1949/02/12. doi: 10.1136/bmj.1.4597.267 ; PMCID: PMCPMC2049513 18109335 PMC2049513

[6] Ma M, Li J, McClane BA. Genotypic and phenotypic characterization of Clostridium perfringens isolates from Darmbrand cases in post-World War II Germany. Infect Immun. 2012;80(12):4354–63. Epub 2012/10/03. doi: 10.1128/IAI.00818-12 ; PMCID: PMCPMC3497428 23027533 PMC3497428

[7] Murrell TGC. Some epidemiological features of pig-bel. 1966. P N G Med J. 2005;48(1–2):27–38. 16894834

[8] Lawrence G, Walker PD, Garap J, Avusi M. The occurrence of Clostridium welchii type C in Papua New Guinea. P N G Med J. 1979;22(1):69–73. 233178

[9] Cooke RA. Changing patterns of disease in Papua New Guinea over a period of 40 years from 1962. Pathology encountered in a stoneage culture by the first western-trained doctors who entered the country. Arkh Patol. 2003;65(1):45–50. Epub 2003/04/03. 12669614

[10] Dwyer PD. People, pigs and parasites in New Guinea: relational contexts and epidemiological possibilities. Parasitol Int. 2006;55 Suppl:S167–73. Epub 2005/12/13. doi: 10.1016/j.parint.2005.11.026 16337181

[11] Lawrence G. The pathogenesis of pig-bel in Papua New Guinea. 1979. P N G Med J. 2005;48(1–2):39–49. 16894835

[12] Toliman PJ, Guwada C, Sou KW. A review of diarrhoea aetiology in Papua New Guinea, 1995-2012. P N G Med J. 2013;56(3–4):145–55. Epub 2013/09/01. 26288933

[13] Lawrence GW, Lehmann D, Anian G, Coakley CA, Saleu G, Barker MJ, et al. Impact of active immunisation against enteritis necroticans in Papua New Guinea. Lancet. 1990;336(8724):1165–7. Epub 1990/11/10. doi: 10.1016/0140-6736(90)92776-e 1978034

[14] Duke T, Myers S, Radcliffe J, Poka H, Deuel K, Miller CM. A surveillance system for pigbel in Papua New Guinea, based on a clinical case definition and laboratory confirmation.. P N G Med J. 2019;62(3–4):114–21.

[15] Poka H, Duke T. In search of pigbel: gone or just forgotten in the highlands of Papua New Guinea?. P N G Med J. 2003;46(3–4):135–42. Epub 2006/02/04. 16454395

[16] Mandrella B. A recent outbreak of necrotizing enteritis in eastern Sri Lanka. Trop Doct. 2007;37(1):52–4. Epub 2007/03/01. doi: 10.1258/004947507779951952 17326895

[17] Petrillo TM, Beck-Sagué CM, Songer JG, Abramowsky C, Fortenberry JD, Meacham L, et al. Enteritis necroticans (pigbel) in a diabetic child. N Engl J Med. 2000;342(17):1250–3. Epub 2000/04/27.doi: 10.1056/NEJM200004273421704 10781621

[18] Gui L, Subramony C, Fratkin J, Hughson MD. Fatal enteritis necroticans (pigbel) in a diabetic adult. Mod Pathol. 2002;15(1):66–70. Epub 2002/01/18. doi: 10.1038/modpathol.3880491 11796843

[19] Matsuda T, Okada Y, Inagi E, Tanabe Y, Shimizu Y, Nagashima K, et al. Enteritis necroticans “pigbel” in a Japanese diabetic adult. Pathol Int. 2007;57(9):622–6. Epub 2007/08/10. doi: 10.1111/j.1440-1827.2007.02149.x 17685936

[20] Wormald JCR, Dindyal S, Mellor F, Behar N. Adult necrotising enterocolitis-pig-bel disease: a Pacific disease in London. BMJ Case Rep. 2016;2016:bcr2016217903. Epub 2016/10/30. doi: 10.1136/bcr-2016-217903 ; PMCID: PMCPMC5093345 27793875 PMC5093345

[21] Chandrasekharam VVSS, Mathur M, Agarwala S, Mitra DK, Bhatnagar V. A clinicopathological study of acute necrotising jejunoileitis. Pediatr Surg Int. 2002;18(5–6):472–6. Epub 2002/11/05. doi: 10.1007/s00383-002-0714-6 12415384

[22] Hannan MJ, Hoque MM. Intestinal obstruction in children due to segmental enteritis: experience in Chittagong, Bangladesh. Pediatr Surg Int. 2012;28(3):277–80. Epub 2011/09/20. doi: 10.1007/s00383-011-2976-3 21928124

[23] Zhao W, Daroca PJ Jr, Crawford BE. Clostridial enteritis necroticans versus secondary clostridial infection superimposed upon ischemic bowel disease. J La State Med Soc. 2002;154(5):251–5. 12440753

[24] De Ruyck E, Baert F, Ghillebert G. An uncommon cause of coffee ground emesis : necrotizing enteritis with pneumatosis intestinalis. Acta Gastroenterol Belg. 2018;81(1):111–2. Epub 2018/03/22. 29562389

[25] Zante B, Tinguley P, Ott D, Dettmer M, Gloor B, Schefold J. Enteritis necroticans - megacolon with massive portal venous gas embolization in a patient after malabsorptive bariatric surgery. Anaesthesiol Intensive Ther. 2019;51(4):333–4. Epub 2019/10/17. doi: 10.5114/ait.2019.88573 31617694

[26] Al Radaideh AJ, Badran EF, Shehabi AA. Diversity of toxin genotypes and antimicrobial susceptibility of Clostridium perfringens isolates from feces of infants. Germs. 2019;9(1):28–34. Epub 2019/05/24. doi: 10.18683/germs.2019.1154 ; PMCID: PMCPMC6446489 31119114 PMC6446489

[27] Cai Y, Gao J, Wang X, Chai T, Zhang X, Duan H, et al. Clostridium perfringens toxin types from freshwater fishes in one water reservoir of Shandong Province of China, determined by PCR. Dtsch Tierarztl Wochenschr. 2008;115(8):292–4–296–7. Epub 2008/08/23. 18717056

[28] Moussa IMI, Hessan AM. Molecular typing of Clostridium perfringens toxins recovered from Central Saudi Arabia. Saudi Med J. 2011;32(7):669–74. 21748201

[29] Park CS, Hwang JY, Cho GJ. The First Identification and Antibiogram of Clostridium perfringens Type C isolated from soil and the feces of dead foals in South Korea. Animals (Basel). 2019;9(8):579. Epub 2019/08/23. doi: 10.3390/ani9080579 ; PMCID: PMCPMC6719196 31434197 PMC6719196

[30] Li J, Sayeed S, McClane BA. Prevalence of enterotoxigenic Clostridium perfringens Isolates in Pittsburgh (Pennsylvania) area soils and home kitchens. Appl Environ Microbiol. 2007;73(22):7218–24. doi: 10.1128/AEM.01075-07 ; PMCID: PMCPMC2168196 17905877 PMC2168196

[31] Hashimoto A, Suzuki H, Oonaka K. Prevalence of cpe-positive Clostridium perfringens in surface-attached soil of commercially available potatoes and its significance as a potential source of food poisoning. Anaerobe. 2023;79:102687. Epub 20221220. doi: 10.1016/j.anaerobe.2022.102687 36549463

[32] Rood JI, Adams V, Lacey J, Lyras D, McClane BA, Melville SB, et al. Expansion of the Clostridium perfringens toxin-based typing scheme. Anaerobe. 2018;53:5–10. Epub 2018/06/06. doi: 10.1016/j.anaerobe.2018.04.011 29866424 PMC6195859

[33] Sayeed S, Uzal FA, Fisher DJ, Saputo J, Vidal JE, Chen Y, et al. Beta toxin is essential for the intestinal virulence of *Clostridium perfringens* type C disease isolate CN3685 in a rabbit ileal loop model. Mol Microbiol. 2008;67(1):15–30. Epub 2007/12/15. doi: 10.1111/j.1365-2958.2007.06007.x 18078439

[34] Fisher DJ, Fernandez-Miyakawa ME, Sayeed S, Poon R, Adams V, Rood JI, et al. Dissecting the contributions of Clostridium perfringens type C toxins to lethality in the mouse intravenous injection model. Infect Immun. 2006;74(9):5200–10. doi: 10.1128/IAI.00534-06 ; PMCID: PMCPMC1594841 16926413 PMC1594841

[35] Shatursky O, Bayles R, Rogers M, Jost BH, Songer JG, Tweten RK. Clostridium perfringens beta-toxin forms potential-dependent, cation-selective channels in lipid bilayers. Infect Immun. 2000;68(10):5546–51. Epub 2000/09/19. doi: 10.1128/IAI.68.10.5546-5551.2000 ; PMCID: PMCPMC101504 10992452 PMC101504

[36] Tweten RK. Clostridium perfringens beta toxin and Clostridium septicum alpha toxin: their mechanisms and possible role in pathogenesis. Vet Microbiol. 2001;82(1):1–9. Epub 2001/06/26. doi: 10.1016/s0378-1135(01)00372-8 11423190

[37] Roos S, Wyder M, Candi A, Regenscheit N, Nathues C, van Immerseel F, et al. Binding studies on isolated porcine small intestinal mucosa and in vitro toxicity studies reveal lack of effect of C. perfringens beta-toxin on the porcine intestinal epithelium. Toxins (Basel). 2015;7(4):1235–52. Epub 2015/04/11. doi: 10.3390/toxins7041235 ; PMCID: PMCPMC4417965 25860161 PMC4417965

[38] Bruggisser J, Tarek B, Wyder M, Müller P, von Ballmoos C, Witz G, et al. CD31 (PECAM-1) serves as the endothelial cell-specific receptor of Clostridium perfringens β-Toxin. Cell Host Microbe. 2020;28(1):69–78.e6. Epub 2020/06/05. doi: 10.1016/j.chom.2020.05.003 32497498

[39] Thiel A, Mogel H, Bruggisser J, Baumann A, Wyder M, Stoffel MH, et al. Effect of Clostridium perfringens β-Toxin on Platelets. Toxins (Basel). 2017;9(10):336. Epub 2017/10/25. doi: 10.3390/toxins9100336 ; PMCID: PMCPMC5666382 29064418 PMC5666382

[40] Tarek B, Bruggisser J, Cattalani F, Posthaus H. Platelet endothelial cell adhesion molecule 1 (CD31) Is essential for Clostridium perfringens Beta-toxin mediated cytotoxicity in human endothelial and monocytic cells. Toxins (Basel). 2021;13(12):893. doi: 10.3390/toxins13120893 ; PMCID: PMCPMC8703487 34941730 PMC8703487

[41] Nagahama M, Hayashi S, Morimitsu S, Sakurai J. Biological activities and pore formation of Clostridium perfringens beta toxin in HL 60 cells. J Biol Chem. 2003;278(38):36934–41. Epub 2003/07/10. doi: 10.1074/jbc.M306562200 12851396

[42] Gurtner C, Popescu F, Wyder M, Sutter E, Zeeh F, Frey J, et al. Rapid cytopathic effects of Clostridium perfringens beta-toxin on porcine endothelial cells. Infect Immun. 2010;78(7):2966–73. Epub 2010/04/21. doi: 10.1128/IAI.01284-09 ; PMCID: PMCPMC2897397 20404076 PMC2897397

[43] Autheman D, Wyder M, Popoff M, D’Herde K, Christen S, Posthaus H. Clostridium perfringens beta-toxin induces necrostatin-inhibitable, calpain-dependent necrosis in primary porcine endothelial cells. PLoS One. 2013;8(5):e64644. Epub 2013/06/05. doi: 10.1371/journal.pone.0064644 ; PMCID: PMCPMC3667183 the authors’ adherence to all the PLOS ONE policies on sharing data and materials. 23734212 PMC3667183

[44] Chen J, Ma M, Uzal FA, McClane BA. Host cell-induced signaling causes Clostridium perfringens to upregulate production of toxins important for intestinal infections. Gut Microbes. 2014;5(1):96–107. Epub 2013/09/26. doi: 10.4161/gmic.26419 ; PMCID: PMCPMC4049945 24061146 PMC4049945

[45] Schäfer K, Wyder M, Gobeli S, Candi A, Doherr MG, Zehnder B, et al. Detection of Clostridium perfringens type C in pig herds following disease outbreak and subsequent vaccination. Vet Rec. 2012;171(20):503. Epub 2012/10/27. doi: 10.1136/vr.101052 23100304

[46] Songer JG. Clostridial enteric diseases of domestic animals. Clin Microbiol Rev. 1996;9(2):216–34. doi: 10.1128/CMR.9.2.216 ; PMCID: PMCPMC172891 8964036 PMC172891

[47] Johnson S, Echeverria P, Taylor DN, Paul SR, Coninx R, Sakurai J, et al. Enteritis necroticans among Khmer children at an evacuation site in Thailand. Lancet. 1987;2(8557):496–500. Epub 1987/08/29. doi: 10.1016/s0140-6736(87)91803-4 2887787

[48] Keyburn AL, Yan X-X, Bannam TL, Van Immerseel F, Rood JI, Moore RJ. Association between avian necrotic enteritis and *Clostridium perfringens* strains expressing NetB toxin. Vet Res. 2010;41(2):21. Epub 20091125. doi: 10.1051/vetres/2009069 ; PMCID: PMCPMC2797654 19931005 PMC2797654

[49] Hawley JH, Peanasky RJ. Ascaris suum: are trypsin inhibitors involved in species specificity of Ascarid nematodes? Exp Parasitol. 1992;75(1):112–8. doi: 10.1016/0014-4894(92)90126-u 1639157

[50] Jäggi M, Wollschläger N, Abril C, Albini S, Brachelente C, Wyder M, et al. Retrospective study on necrotizing enteritis in piglets in Switzerland. Schweiz Arch Tierheilkd. 2009;151(8):369–75. Epub 2009/08/05. doi: 10.1024/0036-7281.151.8.369 19653160

[51] Sakurai J, Duncan CL. Some properties of beta-toxin produced by Clostridium perfringens type C. Infect Immun. 1978;21(2):678–80. Epub 1978/08/01. doi: 10.1128/iai.21.2.678-680.1978 ; PMCID: PMCPMC422049 211090 PMC422049

[52] Macias Rioseco M, Beingesser J, Uzal FA. Freezing or adding trypsin inhibitor to equine intestinal contents extends the lifespan of *Clostridium perfringens* beta toxin for diagnostic purposes. Anaerobe. 2012;18(3):357–60. Epub 2012/04/21. doi: 10.1016/j.anaerobe.2012.03.003 22516562

[53] Davis MW, editor Pigbel: necrotising enteritis in Papua New Guinea 1984. Goroka, Eastern Highlands Province, PNG: Papua New Guinea Institute of Medical Research; 1984.

[54] Weström BR, Svendsen J, Karlsson BW. Protease inhibitor levels in porcine mammary secretions. Biol Neonate. 1982;42(3–4):185–94. Epub 1982/01/01. doi: 10.1159/000241597 6182928

[55] el Newihi H, Dooley CP, Saad C, Staples J, Zeidler A, Valenzuela JE. Impaired exocrine pancreatic function in diabetics with diarrhea and peripheral neuropathy. Dig Dis Sci. 1988;33(6):705–10. doi: 10.1007/BF01540434 2897272

[56] Cucchiara S, Franzese A, Salvia G, Alfonsi L, Iula VD, Montisci A, et al. Gastric emptying delay and gastric electrical derangement in IDDM. Diabetes Care. 1998;21(3):438–43. doi: 10.2337/diacare.21.3.438 9540029

[57] Camilleri M, Malagelada JR. Abnormal intestinal motility in diabetics with the gastroparesis syndrome. Eur J Clin Invest. 1984;14(6):420–7. doi: 10.1111/j.1365-2362.1984.tb01206.x 6441717

[58] Iwanaka T, Kawashima H, Kishimoto H, Kakinuma M, Arai K, Sakurai J, et al. Enteritis necroticans caused by Clostridium perfringens type A. J Pediatr. 2004;144(3):410. Epub 2004/03/06. doi: 10.1016/j.jpeds.2003.09.039 15001960

[59] Hagiya H, Naito H, Sugiyama J, Nojima H, Hagioka S, Morimoto N. Necrotizing duodenitis caused by *Clostridium perfringens* type A in a Japanese young man. Intern Med. 2012;51(20):2973–6. Epub 2012/10/16. doi: 10.2169/internalmedicine.51.8407 23064579

[60] Sobel J, Mixter CG, Kolhe P, Gupta A, Guarner J, Zaki S, et al. Necrotizing enterocolitis associated with *Clostridium perfringens* type A in previously healthy north american adults. J Am Coll Surg. 2005;201(1):48–56. Epub 2005/06/28. doi: 10.1016/j.jamcollsurg.2005.02.029 15978443

[61] Bos J, Smithee L, McClane B, Distefano RF, Uzal F, Songer JG, et al. Fatal necrotizing colitis following a foodborne outbreak of enterotoxigenic *Clostridium perfringens* type A infection. Clin Infect Dis. 2005;40(10):e78–83. Epub 2005/04/22. doi: 10.1086/429829 15844055

[62] Centers for Disease Control and Prevention (CDC). Fatal foodborne *Clostridium perfringens* illness at a state psychiatric hospital--Louisiana, 2010. MMWR Morb Mortal Wkly Rep. 2012;61(32):605–8. Epub 2012/08/17. 22895383

[63] Richard OK, Grahofer A, Nathues H, Posthaus H. Vaccination against *Clostridium perfringens* type C enteritis in pigs: a field study using an adapted vaccination scheme. Porcine Health Manag. 2019;5:20. Epub 20190815. doi: 10.1186/s40813-019-0127-8 ; PMCID: PMCPMC6694488 31428441 PMC6694488

[64] Li D-Y, Scheimann AO, Songer JG, Person RE, Horwitz M, Resar L, et al. Enteritis necroticans with recurrent enterocutaneous fistulae caused by *Clostridium perfringens* in a child with cyclic neutropenia. J Pediatr Gastroenterol Nutr. 2004;38(2):213–5. Epub 2004/01/22. doi: 10.1097/00005176-200402000-00021 14734887

[65] Songer JG, Uzal FA. Clostridial enteric infections in pigs. J Vet Diagn Invest. 2005;17(6):528–36. Epub 2004/01/22. doi: 10.1177/104063870501700602 16475510

[66] Das S, Majumder S, Mathur C, Kingston JJ. Molecular characterization and phylogenetic analysis of *Clostridium perfringens* from animals and their environments by cpn60 UT sequencing analysis. Infect Genet Evol. 2018;58:209–17. Epub 2017/12/27. doi: 10.1016/j.meegid.2017.12.007 29278755

[67] Garcia JP, Anderson M, Blanchard P, Mete A, Uzal FA. The pathology of enterotoxemia by Clostridium perfringens type C in calves. J Vet Diagn Invest. 2013;25(3):438–42. Epub 2013/04/18. doi: 10.1177/1040638713483467 23592750

[68] Diab SS, Kinde H, Moore J, Shahriar MF, Odani J, Anthenill L, et al. Pathology of *Clostridium perfringens* type C enterotoxemia in horses. Vet Pathol. 2012;49(2):255–63. Epub 2011/04/20. doi: 10.1177/0300985811404710 21502373

[69] Uzal FA, Songer JG. Diagnosis of *Clostridium perfringens* intestinal infections in sheep and goats. J Vet Diagn Invest. 2008;20(3):253–65. Epub 2008/05/08. doi: 10.1177/104063870802000301 18460610

[70] Schumacher VL, Martel A, Pasmans F, Van Immerseel F, Posthaus H. Endothelial binding of beta toxin to small intestinal mucosal endothelial cells in early stages of experimentally induced *Clostridium perfringens* type C enteritis in pigs. Vet Pathol. 2013;50(4):626–9. Epub 20120924. doi: 10.1177/0300985812461362 23012387

